# Diabetes Self-management Apps: Systematic Review of Adoption Determinants and Future Research Agenda

**DOI:** 10.2196/28153

**Published:** 2022-07-28

**Authors:** Hessah Alaslawi, Ilhem Berrou, Abdullah Al Hamid, Dari Alhuwail, Zoe Aslanpour

**Affiliations:** 1 Department of Clinical and Pharmaceutical Sciences University of Hertfordshire Hatfield United Kingdom; 2 School of Health & Social Wellbeing University of the West of England Bristol United Kingdom; 3 Saudi Ministry of Health Najran Saudi Arabia; 4 Department of Information Science, College of Computing Sciences and Engineering Kuwait University Kuwait Kuwait

**Keywords:** diabetes self-management, mobile apps, mobile health, mHealth adoption, mobile phone

## Abstract

**Background:**

Most diabetes management involves self-management. Effective self-management of the condition improves diabetes control, reduces the risk of complications, and improves patient outcomes. Mobile apps for diabetes self-management (DSM) can enhance patients’ self-management activities. However, they are only effective if clinicians recommend them, and patients use them.

**Objective:**

This study aimed to explore the determinants of DSM apps’ use by patients and their recommendations by health care professionals (HCPs). It also outlines the future research agenda for using DSM apps in diabetes care.

**Methods:**

We systematically reviewed the factors affecting the adoption of DSM apps by both patients and HCPs. Searches were performed using PubMed, Scopus, CINAHL, Cochrane Central, ACM, and Xplore digital libraries for articles published from 2008 to 2020. The search terms were *diabetes, mobile apps,* and *self-management*. Relevant data were extracted from the included studies and analyzed using a thematic synthesis approach.

**Results:**

A total of 28 studies met the inclusion criteria. We identified a range of determinants related to patients’ and HCPs’ characteristics, experiences, and preferences. Young female patients were more likely to adopt DSM apps. Patients’ perceptions of the benefits of apps, ease of use, and recommendations by patients and other HCPs strongly affect their intention to use DSM apps. HCPs are less likely to recommend these apps if they do not perceive their benefits and may not recommend their use if they are unaware of their existence or credibility. Young and technology-savvy HCPs were more likely to recommend DSM apps.

**Conclusions:**

Despite the potential of DSM apps to improve patients’ self-care activities and diabetes outcomes, HCPs and patients remain hesitant to use them. However, the COVID-19 pandemic may hasten the integration of technology into diabetes care. The use of DSM apps may become a part of the new normal.

## Introduction

### Background

Diabetes prevalence continues to increase worldwide, affecting 1 in 11 people [1]. Persistent hyperglycemia leads to the development of microvascular and macrovascular complications and increases the risk of death; this risk is highest in the young age group [2]. The management of diabetes-induced cardiovascular disease and chronic kidney disease requires heavy health care resource consumption and up to a 4-fold increase in health care costs [3]. Type 2 diabetes is the most prevalent form of this condition and is characterized by persistent hyperglycemia and insulin resistance. Most patients are managed in primary care settings, and given the increasing prevalence, health care settings are experiencing unprecedented demands for clinical appointments and input from health care professionals (HCPs). This often means that patients have limited time with clinicians to discuss diabetes management and optimize treatment [4]. Diabetes self-management (DSM) can improve glycemic control and reduce the risk of complications [5].

Most diabetes management is thought to involve self-management [6]. The term self-management is often used interchangeably with self-care. Self-care refers to behaviors and activities undertaken to manage acute illnesses or injuries, with a focus on treatment [7]. Self-management is a more appropriate term when describing the strategies that patients use to cope with the emotional and practical issues encountered while living with a long-term illness [7]. For patients living with type 2 diabetes, DSM entails adherence to prescribed medication, maintaining a healthy diet, regular physical activity, routine foot checks, frequent monitoring of blood glucose levels if using insulin or sulfonylureas, and managing symptoms of low or very high glucose levels [8]. Patients also have to cope with the reality of diabetic microvascular and macrovascular complications [9] and an increased risk of disability and death [10]. Therefore, DSM education and support is paramount, especially at the point of diagnosis, to influence patients’ behaviors and enhance their engagement with diabetes care [11]. When first diagnosed, patients usually receive DSM education and support from HCPs, followed by ongoing support from other practitioners and community resources [11].

HCPs are increasingly supporting autonomous DSM given the current strain on health care resources [5] and the fact that face-to-face consultations and education courses may not work for everyone. Digital technology has been shown to encourage autonomy and improve diabetes outcomes [12]. Digital and wireless technologies are widely available to support lifestyle and treatment interventions as well as diabetes medical devices, such as blood glucose meters, continuous glucose monitoring devices, and smart insulin pens and pumps [13]. However, mobile health (mHealth) apps for diabetes management are at the forefront of innovations that support DSM. A range of diabetes health apps are available, including nutrition, physical activity, glucose monitoring, insulin titration and delivery, and artificial pancreas systems [13].

Mobile apps have been shown to reduce the barriers to self-management activities, as they provide diabetes education, data logging and trend viewing, and connecting and transferring data to HCPs [14]. Furthermore, mobile apps can be useful elements in effectively modifying lifestyles [15]. The use of apps can lead to a significant reduction in hemoglobin A_1c_ levels among patients with type 2 diabetes [16], improve communication with HCPs, and facilitate remote disease monitoring [17].

### Objectives

Several studies have reported factors that affect patients’ adoption (use) of diabetes management apps, including patients’ characteristics and experiences, app characteristics and functions, and recommendations by HCPs and other patients [18]. Various theoretical lenses have been used to explore app adoption, including the technology acceptance model and the diffusion of innovation theory [19], theory of reasoned action, and unified theory of acceptance and use of technology [20]. However, very few studies examined the antecedents influencing HCPs’ recommendation of DSM apps to their patients and integrating them into their practice [21]. Although many studies have explored the factors that affect patients’ adoption of DSM mobile apps using varying study designs and sample sizes, a systematic overview of these factors and their importance remains missing. Thus, this paper aimed to systematically review the determinants of DSM app adoption by HCPs and patients, highlighting their significance in facilitating or hindering their use. The term adoption will be used throughout to indicate patients’ use of DSM apps and HCPs recommendation of these apps or integrating them in their practice.

This review makes 3 main contributions. First, it provides a comprehensive and systematic review of all studied determinants of DSM app adoption by HCPs and patients. Second, this review highlights the significance of each of these determinants based on the frequency of reporting and the type and sample size of the reporting studies. This will inform commissioners and diabetes app developers of what patients and HCPs look for in DSM apps and the circumstances in which they decide to adopt or reject their use. Third, this review combined patients’ and HCPs’ perspectives on the determinants of DSM app adoption. This is critical because DSM apps can only be effective if HCPs recommend them, and patients use them.

## Methods

### Data Sources and Searches

We searched PubMed, Scopus, CINAHL, ACM digital library, IEEE Xplore digital library and Cochrane Central using the terms “*adoption (uptake, acceptance, use, implement)*,” “*mobile apps (apps, mHealth, smartphones, digital health intervention)*,” and “*T2DM (diabetes mellitus, type 2, chronic conditions, long-term conditions).*” We also checked the references of the selected studies and the references of systematic reviews exploring the use of mobile apps for DSM. Multimedia Appendix 1 [22-49] lists the search strategy used for PubMed. The search strategy for PubMed was adapted to search other databases.

### Eligibility Criteria

We included original studies published between 2008 (when the main app stores, iOS and Android, were launched) and February 2020, which reported on the factors affecting the adoption of self-management apps for diabetes care, involving patients with type 2 diabetes, and HCPs, or stakeholders, or caregivers dealing with patients with diabetes, using quantitative, qualitative, or mixed methods. We did not exclude studies involving patients with type 2 and type 1 diabetes, patients with type 2 diabetes and other comorbidities, or patients who did not specify their diabetes type. This was done to ensure the inclusion of all relevant studies involving patients with type 2 diabetes.

Adoption refers to the decision to proceed with the full or partial implementation of an innovation [50]. In this study, the term adoption specifically refers to patients’ use of DSM apps and HCPs’ recommendation of these apps and integrating them in their practice. Mobile apps are defined as “software applications that can be executed on a mobile platform or a web-based software application that is tailored to a mobile platform but is executed on a server” [51]. Studies on health informatics or digital health intervention or health information technology or telemedicine or telehealth or mHealth have been included in this review if the use of mobile diabetes apps is clearly highlighted. We excluded studies reporting on digital health interventions that did not involve the use of a mobile app, including the use of other mobile functions (eg, calls and SMS).

In all, 2 reviewers (HA and AA) independently screened the titles and abstracts and then full texts to select eligible studies. Reviewers resolved disagreements through discussion or, if necessary, through discussion with an arbitrator (IB).

### Data Extraction and Quality Assessment

Data extraction and quality assessment were performed by HA and verified by IB, and any disagreements were resolved through discussion within the review team. For studies reporting on mHealth in general, including mobile apps, and eHealth in general, including mobile apps, careful extraction of data relating to mobile apps was performed whenever possible. Critical appraisal skill program tools [52] were used for the quality assessment of qualitative studies, cohort studies, and case-control studies. To cover the quality assessment of cross-sectional studies, the Joanna Briggs Institute critical tools for observational studies were used [53]. The quality of the included studies was independently assessed by HA and DA. The reviewers resolved the discrepancies through discussion.

### Data Synthesis and Analysis

To generate new insights from the included studies, the thematic synthesis methodology of Thomas and Harden (2008) [54] was used, as it provides a clear process for synthesizing qualitative data reported in different study designs. This process of data synthesis follows 3 steps: line-by-line coding, organization of *free codes* to build *descriptive* themes and the development of *analytical* themes.

Descriptive data related to the study design, participant type and age, sample size, types of mobile apps used, and study outcomes were extracted. Data pertaining to the factors affecting participants’ use of mobile apps for DSM were independently coded by 2 reviewers (HA and IB). Discrepancies in coding were resolved through discussion and the coding frame was modified accordingly. Similarities between codes were highlighted, and codes were stratified into (descriptive) themes to describe data patterns. This was followed by synthesizing or interrogating descriptive themes to develop analytical themes. Although this method is mainly used to synthesize evidence from qualitative studies, it remains a useful approach for synthesizing qualitative data that can be reported in quantitative studies. In their review of systematic reviews, Hong et al [55] noted that data-based convergent synthesis design was commonly used, where data from qualitative and quantitative studies were analyzed using the same synthesis method, and the results are presented together.

## Results

### Characteristics of the Included Studies

**Figure 1:**
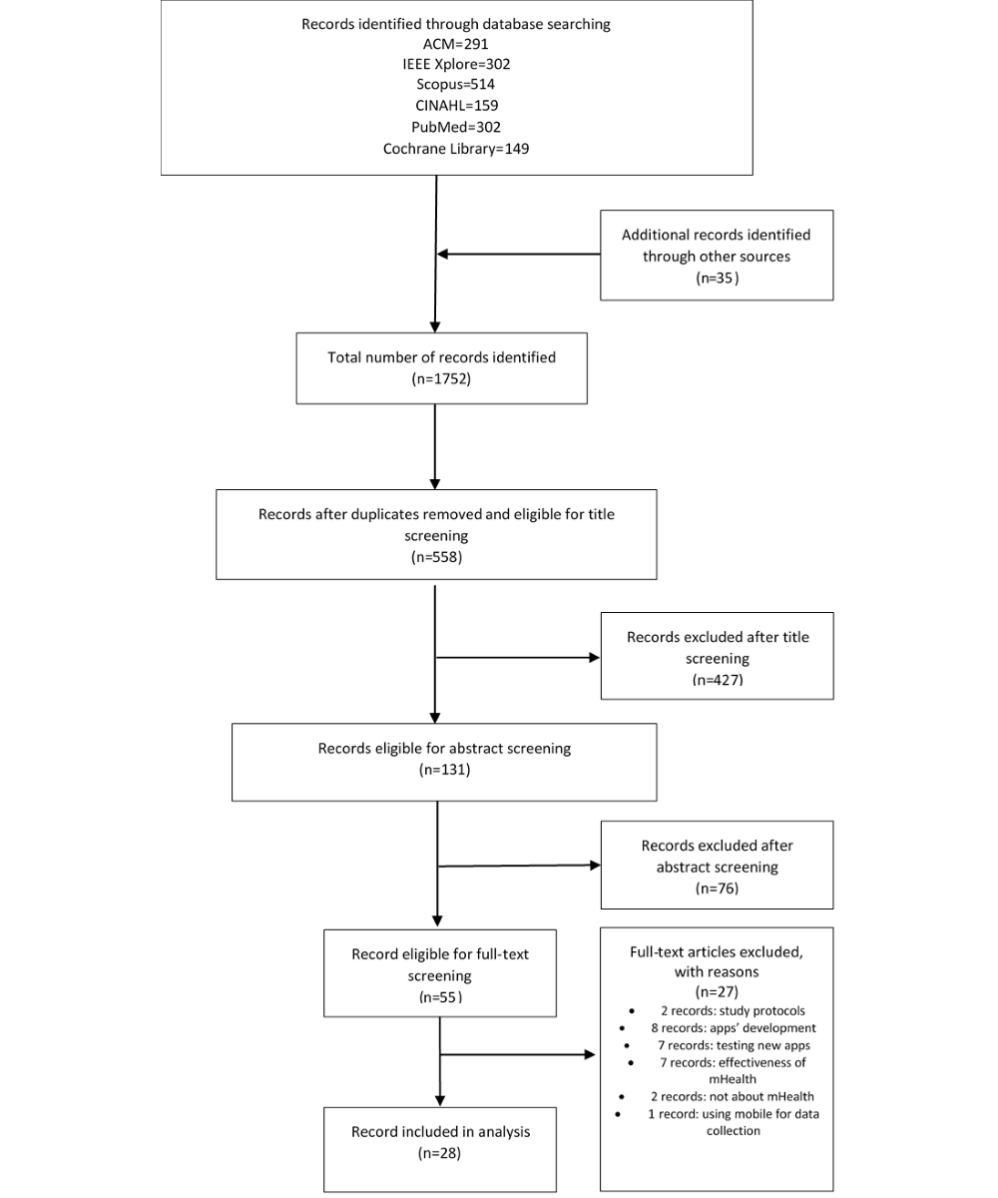
Study selection flow chart.

A total of 28 studies met the inclusion criteria. Figure 1 illustrates the study selection process. We identified 1752 citations from 6 databases (291 articles from ACM, 302 from IEEE Xplore, 514 from Scopus, 302 from PubMed, 149 from Cochrane Library, and 159 from CINAHL). A total of 131 articles passed title screening, and 55 articles passed the abstract screening. From the 55 articles, 27 (49%) articles were eliminated during full-text screening: 2 records were not about mHealth, 2 records were study protocols, 8 records were about app development, 7 records about testing new apps, 7 records were about the impact of mobile apps on diabetes self-management (DSM), and 1 record was about using mobile apps as tools for collecting data. All retrieved articles were published between 2015 and 2019. Most studies (10/28, 36%) were conducted in the United States [21-24,50-55], followed by Canada (3/28, 11%) [25-27] and the United Kingdom (3/28, 11%) [28-30]. In addition, (2/28, 7%) studies were conducted in each of the following countries: Australia [31,32], Saudi Arabia [33,34], and Germany [35,36]. Furthermore, of 28 studies, 1 (4%) study was conducted in each of the following countries: Peru [37], Denmark [38], Rwanda [39], New Zealand [40], Norway [41], and China [42].

The study design of the retrieved papers included qualitative design in 50% (14/28) of the studies [25,26,28,30-32,34,36-38,43-46,48], cross-sectional design in 43% (12/28) of the studies [22,23,27,29,33,35,36,39-42,47,49], cohort design in 4% (1/28) of the studies [24], and mixed methods (cross-sectional design and qualitative design) in 4% (1/28) of the studies [36]. Most studies were primary (26/28, 92%). The data in one study was reported from app entries [24], and another study used secondary data from a national survey [27]. The quality of most included studies was moderate to high (11 and 12, respectively). In all, 18% (5/28) of the studies were of low quality (Multimedia Appendix 1). Most studies were rated as valuable, despite the quality assessment score.

### The Participants’ Characteristics

The participants in 36% (10/28) of the studies included patients with type 2 diabetes mellitus (T2DM) only [26,29,32-34,37-39,45,48], 18% (5/28) of the studies included patients with type 1 diabetes mellitus and T2DM [22,35,43,46,47], and 7% (2/28) of the studies included patients with diabetes mellitus without specifying the type [25,41]. In 11% (3/28) of the studies, patients had chronic conditions, including diabetes [24,27,31], and 11% (3/28) of the studies included patients with diabetes mellitus and cardiovascular disease [30,42,44]. In addition, 14% (4/28) of the studies included patients and HCPs [36,44,47,49]; 4% (1/28) of the studies included patients with diabetes, HCPs, and research assistants [30] and 4% (1/28) of the studies were conducted exclusively with HCPs [23]. The HCPs included in the studies were dietitians, nurses, diabetes educators, community pharmacists, physicians, and podiatrists. A study included HCPs and decision makers [40], and another study included patients with prediabetes or T2DM and family, friends, and HCPs [28].

Most of the included studies (20/28, 71%) recruited <100 participants, 14% (4/28) of the studies had 100 to 500 participants [25,35,41,47], 7% (2/28) of the studies had 500 to 1000 participants [24,39], and 11% (3/28) of the studies recruited >1000 participants [27,42,49].

All studies involved patients aged >18 years, except for a study that involved patients aged <18 years [41]. On average, the patients taking part in the included studies were in their 30s in one study [46], 40s [27] in another study, 50s in studies (9/26, 35%) [25,31,33,34,37,42,45,47,49], and 60s in studies (7/26, 27%) [22,25,29,39,43,44,48]. A total of 4 studies did not report the patients’ age [28,30,35,36], and 3 studies reported a range of patient ages [24,38,41]. For the studies involving HCPs, a study reported the mean age of 38 (SD 6.2) years [44], 4 studies only provided the participants’ age range [23,33,47,49] and one study did not report the age of the participants [36].

### mHealth Interventions

Various mHealth interventions were explored in the reviewed studies. A total of 21 studies examined mHealth apps for diabetes, and 4 studies explored mHealth interventions for diabetes, including mobile apps [22,35,37,40]. In addition, 3 studies explored eHealth interventions for diabetes, including mHealth mobile apps [31,33,45].

Multimedia Appendix 2 [22-49] summarizes the study design, participant characteristics, mHealth interventions used, key outcomes, and determinants of app adoption reported in the included studies.

### Factors Affecting the Adoption of DSM Apps

This part is organized into two main sections: (1) factors affecting patients’ use of DSM apps and (2) factors affecting HCPs’ recommendation of DSM apps. Each section is further divided into subsections. The included studies identified many factors that were facilitators or barriers to adoption, which were weighed against the study design and sample size to highlight the prevalence of the reported factors.

### Factors Affecting Patients’ Use of DSM Apps

The patients’ sociodemographic and diabetes characteristics, perceptions and experiences, and desired app characteristics determine the likelihood of app adoption.

#### The Patient’s Sociodemographic and Diabetes Characteristics

A total of 33% (9/27) of studies found that younger patients were more likely to use DSM apps [22,35,39,41,42,45,47,49]. In addition, 3 studies reported that female patients [35,41,42] and those with a higher level of education were more likely to engage in DSM app use [41,42,49]. Ernsting et al [42] reported that health app users have a higher level of eHealth literacy (the ability to use information technology for health); the higher the eHealth literacy, the more likely patients will adopt DSM apps. A large cross-sectional study by Zhang et al [49], involving 1276 patients revealed that patients with a higher monthly income are more likely to adopt diabetes apps.

Technology use also affects patients’ adoption of DSM apps. A total of 3 studies showed that smartphone users are more likely to use health apps [22,24,35]. Furthermore 8 studies reported that patients who do not know how to use apps or find apps difficult to use were less likely to use DSM apps [25,26,38,39,43-46]. Finally, 5 studies reported that training patients on how to use apps improves their adoption [34,39,41,43,44].

The duration of diagnosis, frequency of blood glucose monitoring and physical activity, and diabetes control affect patients’ adoption of DSM apps. A total of 3 studies reported that newly diagnosed patients were more likely to use DSM apps [32,33,39]. In addition, patients who regularly monitor their blood glucose levels [39] and undertake regular physical activity [42] were more likely to adopt DSM apps. Patients whose diabetes is adequately controlled and who are not experiencing diabetic complications are less likely to adopt DSM apps [38,44]. Table 1 presents the patients’ sociodemographic and diabetes characteristics that affected their use of DSM apps.

**Table 1:** Patients’ sociodemographic and diabetes characteristics (N=5396).

Themes, factors, and definitions				Sample size (participants), n (%)		Study type		Reference
**Patients’ characteristics**								
	**Age:** younger patients are more likely to use DSM^a^ apps							
				12 (0.22)		Qualitative		[45]
				189 (3.5)		Cross-sectional		[47]
				233 (4.32)		Cross-sectional		[35]
				44 (0.82)		Cross-sectional		[33]
				1500 (27.8)		Cross-sectional		[42]
				60 (1.11)		Cross-sectional		[22]
				796 (14.75)		Cross-sectional		[39]
				355 (6.58)		Cross-sectional		[41]
				1276 (23.65)		Cross-sectional		[49]
	**Gender:** female patients are more likely to use DSM apps							
				233 (4.32)		Cross-sectional		[35]
				1500 (27.8)		Cross-sectional		[42]
				355 (6.58)		Cross-sectional		[41]
	**Education:** the higher the level of education, the more engaged is the patient in app use							
				1500 (27.8)		Cross-sectional		[42]
				355 (6.58)		Cross-sectional		[41]
				1276 (23.65)		Cross-sectional		[49]
	**eHealth literacy:** health app users had higher levels of eHealth literacy			1500 (27.8)		Cross-sectional		[42]
	**Monthly income:** patients with higher income are more likely to use DSM apps			1276 (23.65)		Cross-sectional		[49]
	**Technology use**							
		Smartphone users are more interested in using health apps						
			233 (4.32)		Cross-sectional		[35]	
			60 (1.11)		Cross-sectional		[22]	
			503 (9.32)		Cohort		[24]	
		Patients with difficulties in using new technology are less likely to use DSM apps						
			29 (0.54)		Qualitative		[44]	
			30 (0.56)		Qualitative		[38]	
			21 (0.34)		Qualitative		[46]	
			12 (0.22)		Qualitative		[45]	
			287 (5.32)		Qualitative		[25]	
			18 (0.33)		Qualitative		[26]	
			16 (0.3)		Qualitative		[43]	
			796 (14.75)		Cross-sectional		[39]	
		Training on how to use an app improves its adoption						
			29 (0.54)		Qualitative		[44]	
			11 (0.2)		Qualitative		[34]	
			16 (0.3)		Qualitative		[43]	
			355 (6.58)		Cross-sectional		[41]	
			796 (14.75)		Cross-sectional		[39]	
**Diabetes characteristics**								
	**Length of diagnosis:** newly diagnosed patients are more likely to use DSM apps							
				16 (0.3)		Qualitative		[32]
				44 (0.82)		Cross-sectional		[33]
				796 (14.75)		Cross-sectional		[39]
	**Frequent monitoring of blood glucose levels:** patients who frequently monitor sugar levels are more likely to use DSM apps			796 (14.75)		Cross-sectional		[39]
	**Being active:** physically active patients are more likely to use DSM apps			1500 (27.8)		Cross-sectional		[42]
	**Controlled patients:** patients not experiencing problems with diabetes are less likely to use DSM apps							
				29 (0.54)		Qualitative		[44]
				30 (0.56)		Qualitative		[38]

^a^DSM: diabetes self-management.

#### The Patients’ Perceptions and Experiences

A total of 10 studies reported that patients were confident in their DSM without the need for apps, and they did not perceive or were uncertain of the benefits of DSM apps [26,27,32,34,36,38,39,43,47,48]. Interestingly, in 2 smaller qualitative studies, patients reported that they would not use DSM apps, as this puts them in full control of their diabetes and makes them accountable for their behaviors [26,45].

In addition, 2 studies reported that patients would not use DSM apps because they preferred direct and in-person services and interactions [22,45]. However, 5 studies reported that patients are more likely to use DSM apps if recommended by HCPs [26,30,38,41,49], other patients, or the media [49].

Other barriers to the use of DSM apps relate to patients’ experiences with the apps. Patients are less likely to use DSM apps if data entry is onerous [26,32,36,37,43,48] or patients could not integrate the app with daily activities, creating time constraints [26,32,36,43,44]. Patients are less likely to use DSM apps if they are not aware of their existence [26,36,38,39,47]. Table 2 presents the perceptions and experiences that affect patients’ use of DSM apps.

**Table 2:** Patients’ perceptions and experiences (N=3027).

Themes, factors, and definitions				Sample size (participants), n (%)		Study type		Reference
**Patients’ perceptions**								
	**No perceived benefit:** patients are confident without using apps and do not perceive and are uncertain of the benefits of the app in DSM^a^							
				16 (0.53)		Qualitative		[32]
				30 (0.99)		Qualitative		[38]
				9 (0.3)		Qualitative		[36]
				11 (0.36)		Qualitative		[34]
				16 (0.53)		Qualitative		[43]
				18 (0.6)		Qualitative		[26]
				24 (0.79)		Qualitative		[48]
				189 (6.24)		Cross-sectional		[47]
				163 (5.38)		Cross-sectional		[27]
				796 (26.3)		Cross-sectional		[39]
	**Taking charge and accountability:** patients worry that apps put them in full control of their diabetes and make them accountable for their behavior							
				12 (0.4)		Qualitative		[45]
				18 (0.6)		Qualitative		[26]
	**Direct contact:** patients prefer in-person services							
				12 (0.4)		Qualitative		[45]
				60 (1.98)		Cross-sectional		[22]
	**Recommendation**							
		Patients are more likely to use DSM apps if recommended by HCPs^b^						
			30 (0.99)		Qualitative		[38]	
			18 (0.6)		Qualitative		[26]	
			8 (0.26)		Qualitative		[30]	
			355 (11.73)		Cross-sectional		[41]	
			1276 (42.15)		Cross-sectional		[49]	
		Patients are more likely to use DSM apps if recommended by other patients	1276 (42.15)		Cross-sectional		[49]	
		Patients are more likely to use DSM apps if recommended by media	1276 (42.15)		Cross-sectional		[49]	
	**Lack of awareness of existing apps:** patients do not know of existing DSM apps							
				30 (0.99)		Qualitative		[38]
				9 (0.3)		Qualitative		[36]
				18 (0.6)		Qualitative		[26]
				189 (6.24)		Cross-sectional		[47]
				796 (26.3)		Cross-sectional		[39]
**Patients’ experiences**								
	**Data entry:** patients find data entry burdensome							
				16 (0.53)		Qualitative		[45]
				9 (0.3)		Qualitative		[36]
				15 (0.5)		Qualitative		[37]
				18 (0.6)		Qualitative		[26]
				16 (0.53)		Qualitative		[43]
				24 (0.79)		Qualitative		[48]
				355 (11.73)		Cross-sectional		[41]
	**Time constraint:** patients could not integrate the app with daily activities							
				29 (0.96)		Qualitative		[44]
				16 (0.53)		Qualitative		[32]
				9 (0.3)		Qualitative		[36]
				18 (0.6)		Qualitative		[26]

^a^DSM: diabetes self-management.

^b^HCP: health care professional.

### The Desired App Characteristics

Other factors that affect patients’ use of DSM apps relate to the functions and features of these apps. The studies included in this review either evaluated DSM apps with specific functions or reported on patients’ preferred app functions and features that would encourage them to adopt the DSM app and integrate it into their self-management routines. The functions and features are presented in Tables 3 and 4, respectively.

Functions related to nutrition and diet have been reported in 73% (19/26) of studies (tracking diet, calorie counting, and healthy meal recipes) [22,26,27,29,32-35,37-39,41-44,46-49], followed by blood glucose monitoring functions (diaries and reminders to check blood glucose levels) reported in 58% (15/26) of studies [22,26,29,32,33,35,38,39,41,43,46-49], and physical activity functions (tracking, pedometer functions, and reminders to exercise) reported in 54% (14/26) studies [22,27,29,34,35,37-39,41,42,44,46,48,49].

Patients also prefer DSM apps to include medicine management functions such as insulin calculators, tracking medications, and medication reminders, as reported in 13 studies [22,29-31,35,37,38,41,43,44,46,47,49]. Weight management functions were reported in 11 studies [22,27,29,35,37-39,41-43,46], followed by mental health functions in 7 studies, including stress management and emotional support [27,32,37,39,42,44,46]. Appointment reminder preferences were reported in 4 studies [31,38,46,47], and sleep pattern functions were reported in 2 studies [29,42].

Patients are more likely to use DSM apps if they facilitate communication with HCPs (12/26, 46%) [26,30,31,33,34,36,38,41,43,44,48,49] and patients (7/26, 27%) [28,31,35-37,44,49], are visually appealing (10/26, 39%) [26,32,35-38,43,44,46,48], are easy to use (8/26, 31%) [26,30,34,37,38,41,48,49], are easy to understand (1/26, 4%) [43] and easy to access (1/26, 4%) [48], ensure privacy and security (7/26, 27%) [25,30,35,36,41,43,46], provide instant feedback (5/26, 19%) [32,34,37,42,48] and personalized information (2/26, 8%) [26,44], enable goal setting (4/26, 15%) [26,37,42,46], are not costly (5/26, 19%) [24,38,43,48,49], and are available in the patients’ native language (1/26, 4%) [46]. In addition, patients are more likely to use DSM apps if they provide relevant information about diabetes, latest research, and trends (8/26, 31%) [26,31,36-38,43,46,48], increase access to patients’ medical history and notes (3/26, 12%) [22,31,47], and provide information on how to detect and manage hypoglycemia (2/26, 8%) [39,46]. Patients are less likely to use DSM apps if they experience technical problems that cause frequent app crashes (4/26, 15%) [35,38,43,44].

**Table 3:** The desired diabetes self-management apps’ functions (N=21).

App function	Studies, n (%)	References
Nutrition and diet; for example, carbohydrates counting, diet plans, and reference of nutritional values on dishes in restaurants	19 (90.5)	[22,26,27,29,32-35,37-39,41-44,46-49]
Blood glucose monitoring; for example, diabetes diary, blood sugar test reminder, and monitoring hypoglycemia symptoms	15 (71.43)	[22,26,29,32,33,35,38,39,41,43,46-49]
Physical activity; for example, tracking physical activity and exercise plan	14 (66.67)	[22,27,29,34,35,37-39,41,42,44,46,48,49]
Medicines management; for example, insulin dose calculator and medication reminders	13 (61.9)	[22,29-31,35,37,38,41,43,44,46,47,49]
Weight management; for example, tracking weight and weight loss plans	11 (52.38)	[22,27,29,35,37-39,41-43,46]
Mental health; for example, monitoring mood and well-being and social support	7 (33.33)	[27,32,37,39,42,44,46]
Appointments reminders	4 (19.05)	[31,38,46,47]
Sleep pattern	2 (9.53)	[29,42]

**Table 4:** The desired diabetes self-management (DSM) apps’ features (N=5524).

Theme (apps’ features): factors and definitions		Sample size (participants) n (%)	Study type	Reference
**Ease of use**				
	Patients are more likely to use DSM apps if they are easy to use			
		15 (0.27)	Qualitative	[37]
		30 (0.54)	Qualitative	[38]
		18 (0.33)	Qualitative	[26]
		11 (0.2)	Qualitative	[34]
		8 (0.15)	Qualitative	[30]
		24 (0.43)	Qualitative	[48]
		355 (6.43)	Cross-sectional	[41]
		1276 (23.1)	Cross-sectional	[49]
	Patients are more likely to use DSM apps if they are easy to understand	16 (0.29)	Qualitative	[43]
	Patients are more likely to use DSM apps if they are easy to access	24 (0.43)	Qualitative	[48]
**Communication**				
	Patients are more likely to use DSM apps if they enable communication with HCPs^a^			
		29 (0.52)	Qualitative	[44]
		30 (0.54)	Qualitative	[38]
		9 (0.16)	Qualitative	[36]
		18 (0.33)	Qualitative	[26]
		11 (0.2)	Qualitative	[34]
		16 (0.29)	Qualitative	[43]
		8 (0.15)	Qualitative	[30]
		24 (0.43)	Qualitative	[48]
		53 (0.96)	Qualitative	[31]
		44 (0.8)	Cross-sectional	[33]
		355 (6.43)	Cross-sectional	[41]
		1276 (23.1)	Cross-sectional	[49]
	Patients are more likely to use DSM apps if they enable communication and knowledge sharing with other patients			
		29 (0.52)	Qualitative	[44]
		15 (0.27)	Qualitative	[37]
		9 (0.16)	Qualitative	[36]
		31 (0.56)	Qualitative	[28]
		53 (0.96)	Qualitative	[31]
		233 (4.22)	Cross-sectional	[35]
		1276 (23.1)	Cross-sectional	[49]
	Patients are more likely to use DSM apps if they have a social media component			
		31 (0.56)	Qualitative	[28]
		8 (0.15)	Qualitative	[30]
		233 (4.22)	Cross-sectional	[35]
**Feedback:** patients are more likely to use DSM apps if they get real-time feedback				
		16 (0.29)	Qualitative	[32]
		15 (0.27)	Qualitative	[37]
		11 (0.2)	Qualitative	[34]
		24 (0.43)	Qualitative	[48]
		1500 (27.15)	Cross-sectional	[42]
**Customization:** patients are more likely to use DSM apps if they provide personalized or tailored information				
		29 (0.52)	Qualitative	[44]
		18 (0.33)	Qualitative	[26]
**Presentation**				
	Patients are more likely to use DSM apps if they include visual aids or visual effects			
		29 (0.52)	Qualitative	[44]
		16 (0.29)	Qualitative	[32]
		30 (0.54)	Qualitative	[38]
		21 (0.38)	Qualitative	[46]
		9 (0.16)	Qualitative	[36]
		15 (0.27)	Qualitative	[37]
		18 (0.33)	Qualitative	[26]
		16 (0.29)	Qualitative	[43]
		24 (0.43)	Qualitative	[48]
		233 (4.22)	Cross-sectional	[35]
	Patients prefer a clear layout of apps and a suitable font size	30 (0.54)	Qualitative	[38]
**Goal setting:** patients are more likely to use DSM apps if they set up goals				
		21 (0.38)	Qualitative	[46]
		15 (0.27)	Qualitative	[37]
		18 (0.33)	Qualitative	[26]
		1500 (27.15)	Cross-sectional	[42]
**Privacy and security:** patients are more likely to use DSM apps if they ensure data privacy and security				
		21 (0.38)	Qualitative	[46]
		9 (0.16)	Qualitative	[36]
		287 (5.2)	Qualitative	[25]
		16 (0.29)	Qualitative	[43]
		8 (0.15)	Qualitative	[30]
		233 (4.22)	Cross-sectional	[35]
		355 (6.43)	Cross-sectional	[41]
**Cost:** patients consider the cost of apps when deciding to use DSM apps				
		503 (9.11)	Cohort	[24]
		30 (0.54)	Qualitative	[38]
		16 (0.29)	Qualitative	[43]
		24 (0.43)	Qualitative	[48]
		1276 (23.1)	Cross-sectional	[49]
**Technical problems:** patients are less likely to use DSM apps if they experience technical problems or app crashes				
		29 (0.52)	Qualitative	[44]
		30 (0.54)	Qualitative	[38]
		16 (0.29)	Qualitative	[43]
		233 (4.22)	Cross-sectional	[35]
**Language:** patients are more likely to use apps if they are in their native language in addition to English		21 (0.38)	Qualitative	[46]
**Information**				
	Information about diabetes and the latest research findings			
		30 (0.54)	Qualitative	[38]
		21 (0.38)	Qualitative	[46]
		9 (0.16)	Qualitative	[36]
		15 (0.27)	Qualitative	[37]
		18 (0.33)	Qualitative	[26]
		16 (0.29)	Qualitative	[43]
		24 (0.43)	Qualitative	[48]
		53 (0.96)	Qualitative	[31]
	Patient information, medical history, and medical notes			
		53 (0.96)	Qualitative	[31]
		189 (3.42)	Cross-sectional	[47]
		60 (1.09)	Cross-sectional	[22]
	Information about symptoms of hypoglycemia and its management			
		21 (0.38)	Qualitative	[46]
		796 (14.41)	Cross-sectional	[39]

^a^HCP: health care professional.

### Factors Affecting HCPs’ Recommendation of DSM Apps

Only a small number of studies involved HCPs [23,28,30,40,44,47,49], despite their role in promoting and facilitating DSM. Table 5 presents the relevant findings.

Some factors identified by patients as determinants of DSM app adoption have also been reported by HCPs. These include patients’ characteristics, beliefs, and experiences. HCPs reported that patients who find it difficult to use or access technology are less likely to use DSM apps, and HCPs will be reluctant to recommend DSM apps to those patients [23,30,44]. Furthermore, HCPs are more likely to recommend DSM apps if they are easy to use [23,30], easy to access [23], provide prompt real-time feedback [30], improve communication between patients and HCPs [49], are free of charge [23,49], and are available in the patients’ language [23]. HCPs also reported in the study by Zhang et al [49] that patients do not trust diabetes apps, and hence, will not be using them and that patients are less likely to use DSM apps if they require onerous and time-consuming data entry tasks.

Similar to patients’ reports, HCPs would recommend DSM apps if they provide information about diabetes and the latest research findings [30]. Other similar factors include the desired functions, features, and information of the apps. Similar to patients, HCPs would recommend DSM apps if they include nutrition and diet functions [23,47], blood glucose monitoring [23,49], physical activity tracking [23], medicines’ management [47], and weight management [23].

HCPs characteristics, beliefs, and awareness of existing DSM apps also affect their recommendation to patients. A study reported that HCPs aged between 40 and 49 years are most likely to recommend DSM apps, and awareness of diabetes apps increases with the HCP’s age [49]. Moreover, HCPs with Master of Science degrees, those registered as dietitian nutritionists [23], and those working in tertiary care settings [49] are more likely to recommend apps to patients. HCPs who routinely use apps are more likely to recommend apps to their patients. Those who are not *technology savvy* are likely to require training sessions on how to use apps before recommending them [23]. Zhang et al [49] suggested that HCPs are not convinced of the impact of DSM apps on blood glucose levels; therefore, they may be reluctant to recommend them. Furthermore, HCPs’ lack of awareness of existing or appropriate DSM apps hinders their recommendations to patients [23,49].

Other factors that may hinder HCPs’ recommendation of app use are related to work pressure. A total of 3 studies highlighted that the heavy workload of HCPs would prevent them from recommending apps, given that they lack the time needed to train patients on how to use the app [23,30,44,49]. HCPs reported in the study by Zhang et al [49] that they may not recommend diabetes apps to patients, as it is not clear if it is legal to provide diabetes care through apps and how to bill the patient for this internet-based care.

**Table 5:** Summary of the factors affecting health care professionals’ (HCPs) recommendations of diabetes self-management (DSM) apps (N=1297).

Themes, factors, and definitions					Sample size (participants), n (%)		Study type		Reference
**Patients’ characteristics—technology use:** HCPs report that patients who face difficulties in using or accessing to technology are less likely to use DSM apps and less likely to recommend apps for them									
					5 (0.39)		Qualitative		[44]
					6 (0.46)		Qualitative		[30]
					583 (44.95)		Cross-sectional		[23]
**Patients’ beliefs—patients’ distrust:** HCPs reported that the main obstacle to use apps is patients’ distrust of the apps					608 (46.88)		Cross-sectional		[49]
**Patients’ experiences**									
	**Data entry:** HCPs report that the patients may find data entry burdensome			6 (0.46)		Qualitative		[30]	
	**Time constraint:** HCPs report that using apps could be time consuming for patients			583 (44.95)		Cross-sectional		[23]	
**HCPs characteristics**									
	**Age:** HCPs awareness about apps increases with age; HCPs aged between 40 and 49 years are more likely to recommend apps for patients			608 (46.88)		Cross-sectional		[49]	
	**Educational levels:** HCPs with masters’ degree and registered dietician nutritionists are more likely to recommend apps for patients			583 (44.95)		Cross-sectional		[23]	
	**Clinical settings:** HCPs in tertiary care are more likely to recommend and use DSM apps for patients			608 (46.88)		Cross-sectional		[49]	
	**Technology use:** HCPs who are not technology savvy require more training about apps								
				5 (0.39)		Qualitative		[44]	
				583 (44.95)		Cross-sectional		[23]	
**HCPs beliefs—no perceived benefits:** HCPs are less likely to recommend apps because of the lack of evidence about their effectiveness					608 (46.88)		Cross-sectional		[49]
**HCPs awareness—lack of awareness**									
			HCPs do not know of the existing apps						
					95 (7.32)		Cross-sectional		[36]
					608 (46.88)		Cross-sectional		[49]
			HCPs do not know about the suitable apps to recommend		608 (46.88)		Cross-sectional		[49]
**Work pressures**									
	**Legal issues:** HCPs are less likely to recommend apps for managing diabetes because they do not know if it is legal to use apps to manage patients			608 (46.88)		Cross-sectional		[49]	
	**Workload:** workload and workflow challenges are the main barriers to recommend DSM apps								
				5 (0.39)		Qualitative		[44]	
				6 (0.46)		Qualitative		[30]	
				608 (46.88)		Cross-sectional		[49]	
	**Billing issues:** uncertainty on how to bill the patients about health care provided through the apps			608 (46.88)		Cross-sectional		[49]	
**Apps features**									
	**Ease of use**								
		HCPs are more likely to recommend DSM apps to patients if they are easy to use							
				6 (0.46)		Qualitative		[30]	
				583 (44.95)		Cross-sectional		[23]	
		HCPs are more likely to recommend DSM apps to patients if it they are easy to access		583 (44.95)		Cross-sectional		[23]	
	**Feedback:** HCPs are more likely to recommend DSM apps to patients if they provide real-time feedback			6 (0.46)		Qualitative		[30]	
	**Communication:** HCPs are more likely to recommend DSM apps to patients if they improve communication with HCPs			608 (46.88)		Cross-sectional		[49]	
	**Cost:** HCPs are more likely to recommend DSM apps to patients if apps are free of charge								
				583 (44.95)		Cross-sectional		[23]	
				608 (46.88)		Cross-sectional		[49]	
	**Multi-language:** HCPs are less likely to recommend DSM apps for patients if apps are not available in the patients’ language			583 (44.95)		Cross-sectional		[23]	
**Apps’ information provision:** HCPs would like the apps to have information about diabetes and new research findings					6 (0.46)		Qualitative		[30]

## Discussion

### Principal Findings

This study systematically reviewed the determinants of DSM app use by patients and their recommendations by HCPs, highlighting their prevalence and significance in facilitating and hindering their uptake. To our knowledge, this is the first review exploring the prevalence and determinants of use by patients with T2DM and HCPs’ recommendations of mobile apps for DSM.

Patients’ sociodemographic characteristics are determinants of app use in DSM. Age has been consistently reported to be a key influencing factor. Younger [56-59], female [60,61] patients were more likely to use DSM apps. Older patients are less likely to engage in digital technologies and health apps [62]. However, the current COVID-19 pandemic highlights that, when necessary, older patients can effectively interact with mobile apps that are beneficial and meet their needs, such as social networking apps and digital health apps [63]. Older patients are an important population to target to improve DSM behaviors [64], given the high prevalence of this condition among this group. Notably, the literature often focuses on biological age as a factor and the assumed decline in cognitive function, sight, hearing, and motor skills over time. However, when considering technology adoption, the concept of age should be expanded to incorporate the *technological age* of patients; people who are aged 60 years in 2020 have had at least 20 years of familiarity or experience with digital technology [65].

Patients’ use of DSM apps is also influenced by their level of education, eHealth literacy, perceptions and digital experiences, and technical skills [56,66-71]. Interestingly, the duration of diagnosis also affected the use of DSM apps. Newly diagnosed patients are more likely to use DSM apps, as shown in the qualitative study by Baptista et al [71]. The authors further clarified that patients may become frustrated with the *basic* content of the apps as they become more experienced with diabetes management.

Direct recommendations by health professionals have been suggested as a significant influencer of patients’ use of DSM apps [72]. However, only a few studies have explored diabetes HCPs’ recommendation of DSM apps and their integration into care pathways. Clinicians are still apprehensive about recommending DSM apps, especially that consensus regarding the strength of their evidence base and evaluation methods is yet to be reached [73].

Several determinants related to DSM apps reported in our review were also postulated as constructs of the main adoption theories; for example, diffusion of innovation theory [74], technology acceptance model [75], and the unified theory of acceptance and use of technology [76]. These include the relative advantages of apps in DSM, compatibility with daily schedules, and ease of use.

It was found that patients with type 2 diabetes prefer interactive apps with functions that aid them in maintaining a healthy lifestyle, reducing weight, and managing their medicines. Privacy, security, and costs also affect use. These are in line with the findings of the review by Adu et al [77] for developing diabetes apps and the review of diabetes-related applications by Doyle-Delgado and Chamberlain [78], as well as the reviews for other health conditions such as hypertension [79], gestational diabetes [80], and chronic conditions [81]. Interestingly, mental health functions were desired to be part of diabetes apps rather than separate or generic apps, which highlights the importance patients assign to integrated mental and diabetes health care.

Studies exploring HCPs’ use and recommendations of DSM apps are scarce. Our review identified similar factors affecting HCPs’ recommendations of DSM to their patients. HCPs are a diverse group of technology users, and their own characteristics and experiences with mobile apps affect their likelihood of recommending these apps [82]. This highlights the need to integrate digital health education into health care curricula [82]. Furthermore, workload pressures [19,66,67] have also been reported to hinder HCPs’ recommendation of apps, especially if time is required to train patients. It is important to consider that because of the lack of regulatory frameworks, digital health clinical guidelines, institutional review, and validation of available apps, HCPs are likely to hesitate to recommend them [13,83].

### Future Research

Looking forward, there are a few issues to consider, especially that digital health apps are likely to be one of the legacies of the COVID-19 pandemic, disrupting traditional health care delivery models [84]. First, researchers have investigated the role and effectiveness of these apps as stand-alone or complementary resources. Efforts should be dedicated to investigate how DSM apps can be integrated into care pathways [83,85], and to explore the roles and responsibilities of health care organizations, HCPs, and patients in a system where DSM apps put the patient in *the driver seat* of managing their condition, the HCP holding the *map* and providing feedback and monitoring, and health care organizations ensuring *road safety* and clinical governance. Furthermore, it is important to explore the impact of ethnicity and race on engagement with and access to diabetes care when mHealth apps and technologies are integrated into care pathways. Mobile apps and technologies may improve access but may also exacerbate inequalities [56]. Answering this question is paramount for designing effective, efficient, and equitable services. It is also important to fully investigate the impact of health care delivery, via mobile apps, on clinical and patient outcomes and how reimbursement and remuneration can be claimed [86].

Second, several ethical issues must be explored when integrating health technologies such as mobile apps into care pathways. One of the most frequently reported barriers to mobile app adoption in health care is the fear of losing human interaction between the patient and the HCP, but at the same time, patients and HCPs see the potential for mobile apps to increase their contact and meaningful input, albeit internet-based. Research could explore how mobile apps can be integrated into care pathways without dehumanizing patients or HCPs [87]. This may warrant investigating how to affect cultural change, especially in relation to the management of long-term conditions and where health technologies fit in the new normal. Privacy is another issue that is often reported when digital technologies are used to deliver health care services. Research could explore the required legal changes, depending on culture and context, to facilitate a safe transfer of information between patients, health care organizations, and relevant stakeholders (and who those stakeholders might be) [88].

Third, regulatory, clinical, and professional bodies’ evaluation and support of apps is a key facilitator to encourage health care organizations and HCPs to recommend apps for patient care and for patients to engage with the recommended apps [13]. Research could develop evaluation and implementation frameworks and inform the development of clinical and care guidelines that integrate mobile apps into disease management pathways.

### Study Strengths and Limitations

This is the first systematic review to present a synthesis of the determinants that affect patients’ use of DSM apps and HCPs recommending them. It also highlights the features and functions required for DSM apps. It draws from a range of studies with qualitative and quantitative designs to improve our understanding of the significance of these factors when deciding to use or recommend a DSM app. However, several potential limitations should be considered when interpreting the findings of this study. First, we included only studies published in peer-reviewed journals, and some of which were of poor quality. Further insights may be reported in conference proceedings and gray literature resources, which were excluded from this study. Second, we included studies that reported on the use of DSM apps in type 2 diabetes, even if those studies reported other types of diabetes or other long-term conditions. This meant that, occasionally, it was not possible to separate data relating to type 2 diabetes from data relating to type 1 diabetes, cardiovascular disease, and other comorbidities. Third, considering the factors reported in this review were not always explicitly highlighted in the included studies, our identification, interpretation, and coding techniques may have affected the review findings. Finally, several of the reported factors are based on what would influence patients and HCPs’ *hypothetical* adoption of DSM apps rather than actual use. Therefore, hypothetical bias must be considered when interpreting the findings of our review.

### Conclusions

DSM is paramount for improving diabetes outcomes and reducing the risk of complications. Mobile apps can facilitate self-management activities if patients use them and HCPs recommend them. Addressing the technology, patient, and HCP factors that may hinder the use of DSM apps can improve their role in diabetes care, especially if these apps are integrated into diabetes care pathways.

## Appendix

Multimedia Appendix 1 PubMed search strategy and the results of the quality assessment of the included studies.

Multimedia Appendix 2 Summary and characteristics of the included studies.

## Abbreviations

DSM: diabetes self-management
HCP: health care professional
mHealth: mobile health
T2DM: type 2 diabetes mellitus
