# Supplementary material for: Diabetes Self-management Apps: Systematic Review of Adoption Determinants and Future Research Agenda
Source: JMIR Diabetes. 2022 Jul 28;7(3):e28153. doi: 10.2196/28153 (PMC9377471; doi:10.2196/28153)
Supplement: Multimedia Appendix 1 [file diabetes_v7i3e28153_app1.docx]

## Multimedia Appendix 1

### PubMed search strategy (6 February 2020)


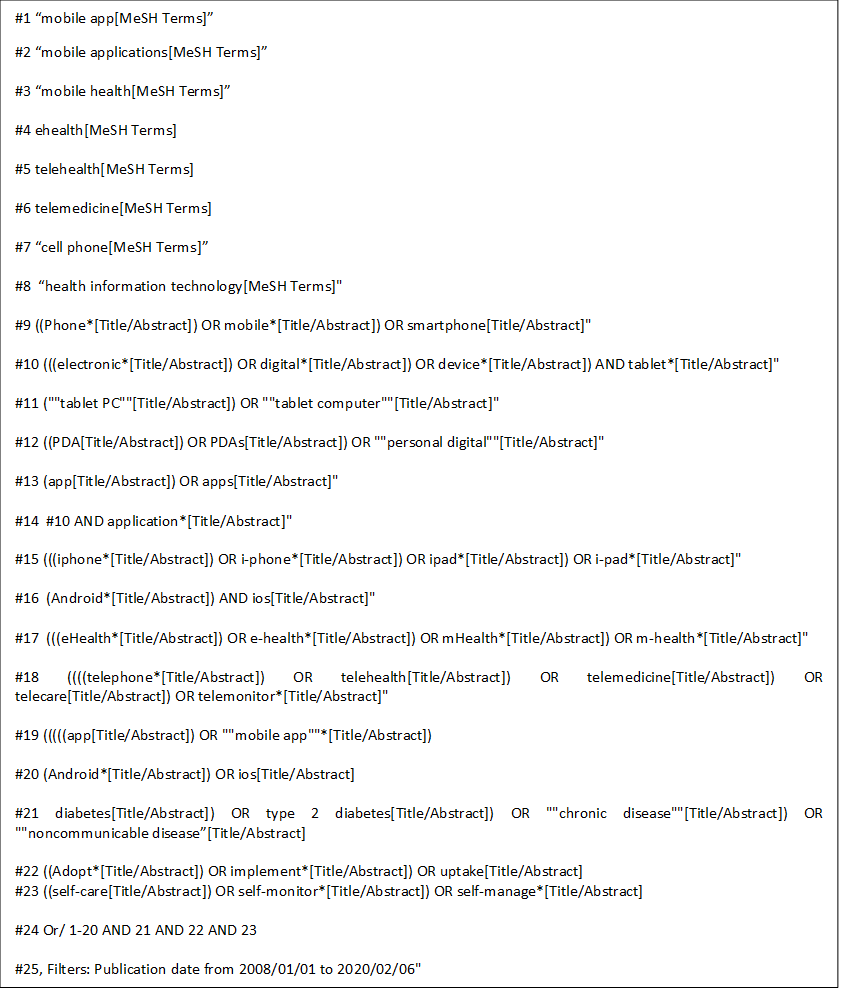


### Quality assessment of the included studies

### Online supplementary table 1: Quality assessment of cross-sectional studies

| **Criteria / Author (year)** | **[40]** | **[47]** | **[35]** | **[33]** | **[42]** | **[22]** | **[23]** | **[36]** | **[41]** | **[27]** | **[29]** | **[39]** | **[49]** |
| --- | --- | --- | --- | --- | --- | --- | --- | --- | --- | --- | --- | --- | --- |
| 1. Were the criteria for inclusion in the sample clearly defined? | Yes | Yes | Yes | Yes | Yes | Yes | Yes | Yes | No | Yes | Yes | Yes | Yes |
| 1. Were the study subjects and the setting described in detail? | Yes | Yes | Yes | Yes | Yes | Yes | Yes | Yes | Yes | Yes | Yes | Yes | Yes |
| 1. Was the exposure measured in a valid and reliable way? | Unclear | Yes | Yes | Yes | Yes | Yes | Yes | Unclear | Unclear | Yes | Yes | Yes | Yes |
| 1. Were objective, standard criteria used for measurement of the condition? | Yes | Yes | Yes | Yes | Yes | Yes | Yes | Unclear | Yes | Yes | Yes | Yes | Yes |
| 1. Were confounding factors identified? | No | Yes | No | Yes | Yes | Yes | Yes | Unclear | No | Yes | Unclear | Yes | No |
| 1. Were strategies to deal with confounding factors stated? | No | Yes | No | Yes | Yes | Yes | Yes | Unclear | No | Yes | Unclear | Yes | No |
| 1. Were the outcomes measured in a valid and reliable way? | Yes | Yes | Yes | Yes | Yes | Yes | Yes | Unclear | Yes | Yes | Yes | Yes | Yes |
| 1. Was appropriate statistical analysis used? | Unclear | Yes | Yes | Yes | Yes | Yes | Yes | No | Yes | Yes | Yes | Yes | Yes |
| Total | 4/8 | 8/8 | 6/8 | 8/8 | 8/8 | 8/8 | 8/8 | 2/8 | 4/8 | 8/8 | 6/8 | 8/8 | 6/8 |

### Online supplementary table 2: Quality assessment of the cohort study

| Criteria/ Author (year) | [24] |
| --- | --- |
| 1. Were the two groups similar and recruited from the same population? | Yes |
| 1. Were the exposures measured similarly to assign people to both exposed and unexposed groups? | Yes |
| 1. Was the exposure measured in a valid and reliable way? | Unclear |
| 1. Were confounding factors identified? | Unclear |
| 1. Were strategies to deal with confounding factors stated? | Unclear |
| 1. Were the groups/participants free of the outcome at the start of the study (or at the moment of exposure)? | Unclear |
| 1. Were the outcomes measured in a valid and reliable way? | Yes |
| 1. Was the follow up time reported and sufficient to be long enough for outcomes to occur? | Not applicable |
| 1. Was follow up complete, and if not, were the reasons to loss to follow up described and explored? | Not applicable |
| 1. Were strategies to address incomplete follow up utilized? | Not applicable |
| 1. Was appropriate statistical analysis used? | Yes |
| Total | 4/11 |

### Online supplementary table 3: Quality assessment of qualitative studies

| Criteria/ Author (year) | [44] | [32] | [46] | [36] | [37] | [38] | [45] | [25] |
| --- | --- | --- | --- | --- | --- | --- | --- | --- |
| 1. Is there congruity between the stated philosophical perspective and the research methodology? | Yes | Unclear | Yes | Unclear | No | Yes | Unclear | Yes |
| 1. Is there congruity between the research methodology and the research question or objectives? | Yes | Yes | Yes | Yes | Yes | Yes | Yes | Yes |
| 1. Is there congruity between the research methodology and the methods used to collect data? | Yes | Yes | Yes | Yes | Yes | Yes | Yes | Yes |
| 1. Is there congruity between the research methodology and the representation and analysis of data? | Yes | Yes | Yes | Unclear | Yes | Yes | Yes | Yes |
| 1. Is there congruity between the research methodology and the interpretation of results? | Yes | Yes | Yes | Yes | Yes | Yes | Yes | Yes |
| 1. Is there a statement locating the researcher culturally or theoretically? | Yes | Yes | Yes | No | No | Yes | Unclear | Yes |
| 1. Is the influence of the researcher on the research, and vice- versa, addressed? | No | Yes | No | No | No | No | Yes | No |
| 1. Are participants, and their voices, adequately represented? | Yes | Yes | Yes | Yes | Yes | Yes | Yes | Unclear |
| 1. Is the research ethical according to current criteria or, for recent studies, and is there evidence of ethical approval by an appropriate body? | Yes | Yes | Yes | Yes | Yes | Yes | Yes | Yes |
| 1. Do the conclusions drawn in the research report flow from the analysis, or interpretation, of the data? | Yes | Yes | Yes | Yes | Yes | Yes | Yes | Yes |
| Total | 9/10 | 9/10 | 9/10 | 6/10 | 7/10 | 9/10 | 8/10 | 8/10 |

| Criteria/ Author (year) | [26] | [34] | [43] | [28] | [48] | [30] | [31] |
| --- | --- | --- | --- | --- | --- | --- | --- |
| 1. Is there congruity between the stated philosophical perspective and the research methodology? | Unclear | Unclear | Yes | No | Yes | Yes | Yes |
| 1. Is there congruity between the research methodology and the research question or objectives? | Yes | Yes | Yes | Yes | Yes | Yes | Yes |
| 1. Is there congruity between the research methodology and the methods used to collect data? | Yes | Yes | Yes | Yes | Yes | Yes | Yes |
| 1. Is there congruity between the research methodology and the representation and analysis of data? | Yes | Yes | Yes | Yes | Yes | Yes | Yes |
| 1. Is there congruity between the research methodology and the interpretation of results? | Yes | Yes | Yes | Yes | Yes | Yes | Yes |
| 1. Is there a statement locating the researcher culturally or theoretically? | Unclear | Unclear | Unclear | No | Yes | Unclear | Unclear |
| 1. Is the influence of the researcher on the research, and vice- versa, addressed? | No | No | Unclear | No | Yes | Unclear | Unclear |
| 1. Are participants, and their voices, adequately represented? | Yes | Yes | Yes | Yes | Yes | Yes | Yes |
| 1. Is the research ethical according to current criteria or, for recent studies, and is there evidence of ethical approval by an appropriate body? | Yes | Yes | Yes | Yes | Yes | Yes | Yes |
| 1. Do the conclusions drawn in the research report flow from the analysis, or interpretation, of the data? | Yes | Yes | Yes | Yes | Yes | Yes | Yes |
| Total | 7/10 | 7/10 | 8/10 | 7/10 | 10/10 | 8/10 | 8/10 |
