# Supplementary material for: Diabetes Self-management Apps: Systematic Review of Adoption Determinants and Future Research Agenda
Source: JMIR Diabetes. 2022 Jul 28;7(3):e28153. doi: 10.2196/28153 (PMC9377471; doi:10.2196/28153)
Supplement: Multimedia Appendix 2 [file diabetes_v7i3e28153_app2.docx]

## Multimedia appendix 2

Table1: Summary and characteristics of the included studies.

| Study,  Location | Aims | Design | Type of participants | Participants number/ gender | Age (years) | Type of mHealth/ software platform | Key findings | Factors affecting adoption |
| --- | --- | --- | --- | --- | --- | --- | --- | --- |
| [40]  KSA | Identify the barriers to mHealth for diabetes care and the relevant solutions | Cross-sectional | Senior clinicians, leading HCPs and decision makers | 30  Male: 24 | 20-30yr: 2  31-40yr: 16  41-50yr: 11  >50yr: 4 | mHealth for diabetes, including: Mobile computing e.g. apps and medical sensors | The main barriers identified in this study were shortage of mHealth expertise, financial issues, legalisation and organizational barriers. The main solutions for barriers were creation of appropriate leadership and clinical environment. | Barriers to implementation: mHealth expertise and human shortage (90.9%), funding and infrastructure investment (87.9%), legal practice standardisation and regulatory barriers (69.7%); and healthcare organizational and bureaucracy barriers (81.8%).  Facilitators to implementation: Provide educational training in mHealth area (94%), better public awareness programmes on the benefit of mHealth (94%), allocation of more funding and strategic plans (82%), set interoperability strategy for digital solutions (97%), set a national mHealth plan (94%), better allocation of resources (91%), development of ethical standards (91%) and creation of mHealth leaders (91%). |
| [44]  Peru | Explore the experience of patients using emotional control app and explore the experience of nurse-support apps’ users | Qualitative | Patients with DM and/or HTN  Nurses who support the apps | Patients  29  Male: 9  Patients with DM: 9  Patients with DM+HTN: 13  Patients with HTN: 7  Nurses  6  Male: 0 | Patients  60 ± 9.6  Nurses  38 ± 6.2 | CONEMO (emotional control): is a technology-driven, psychoeducational, and nurse-supported intervention delivered via a smartphone app, aimed to reduce depressive symptoms in people with diabetes or diabetes and hypertension | Using nurse-supported app to reduce depressive symptoms is possible and mostly perceived beneficial by patients. Addressing barriers by patients and nurses provided valuable information for building mobile health interventions for depression. | Patients  Barriers to using apps: Difficulties in using apps or smartphone; difficulties in opening pages, fear of mobile stolen; low battery duration; difficulties in entering password to unlock screen, memory problems; time and economic constraints; health status was well; prefer face-to-face contact.  App’s features and functionalities: Tailored information, interventions and advices; videos and audio within the app; reminders to follow diet, exercise and medications; information about healthy diet and NCD; less repetitive information; communication with HCPs; communication with other patients.  Nurses  Barriers to support patients via apps: Difficulties in consolidating apps’ activities with their workflow; increased workload. |
| [47]  New Zealand | Explore uses and beliefs about mobile phone apps for diabetes self-management | Cross-sectional | Patients with DM; HCPs who treat DM | Patients: 189  Male: 108  T2DM: 83/189  HCPs: 115  Male: NP | Patients: 50.0 ±15.7  HCPs:  21-30yr: 9  31-40yr: 21  41-50yr: 34  51-60yr: 42  +60yr: 6 | DSM mobile apps | App users were younger; Recording blood glucose was the more favourable function by patients with interest in insulin dose calculating function; HCPs were more confident to recommend glucose and dietary diaries for patients instead of insulin calculators. | Patients  Demographics: Diabetes apps users mean age (39±11.1) is younger than non-users (52.6±15.6)  Barriers to use apps: lack of awareness about existence of apps (79/118), confident without using app (20/118), negative previous experience (20/118).  App features and functionalities (based of users’ preference who currently use apps): diary of blood glucose levels (7/7), meals diary/intake (2/7), Reminders to check blood glucose (3/7), insulin dose calculators (2/7), blood glucose level guidelines (2/7), Personal details and condition information (2/7), calendar of appointments (2/7), contact details of your diabetes team (2/7), dietary advice (1/7).  Requirements for future apps (based on opinions of current users): insulin dose calculators (3/7), meals diary/intake (3/7), Reminders to check blood glucose (3/7), contact details of your diabetes team (3/7), blood glucose level guidelines (2/7), dietary advice (2/7), Personal details and condition information (2/7), calendar of appointments (1/7), diary of blood glucose levels (1/7).  Apps features and functionalities (based on non-app users): diary of blood glucose levels (46/76), meals diary/intake (33/76), Reminders to check blood glucose (36/76), insulin dose calculators (34/76), blood glucose level guidelines (29/76), Personal details and condition information (24/76), calendar of appointments (43/76), contact details of your diabetes team (36/76), dietary advice (30/76).  HCPs:  Dietitians were most likely to have recommended an app (10/12) followed by nurses (42/64).  Apps features and functionalities: Blood glucose diary and meal intake diary, reminders for medications and remainders for checking blood glucose, information, guidelines and insulin calculators.  HCPs confident to recommend apps about: Glucose diaries, reminders, education, insulin calculators. |
| [35]  UK | Assess levels of engagement with mHealth technologies, identify demographic sub-groups that are more likely to use apps and identify features that are most desirable by patients with diabetes. | Cross sectional | Patients with T1DM and T2DM | 230 patients;  Male: 145 T2DM: 139 | - | DSM mobile app | The integration of mHealth technologies has the potential to empower patients, increase patient choice, improve outcomes and provide services; the majority (70.1%) expressed an interest in the use of apps to manage diabetes, however, patients’ preference of apps characteristics and functions does not currently meet the existed apps in the market; user engagement could improve future app development. | Demographics: Younger ages (less or equals to 56) and female gender are more likely to express a preference for apps to help in diabetes.  Apps features and functionalities: Data security and password protection (89/129); enable health records, data and email back-up, social media integration (26/131), visual presentation (40-50%), glucose monitoring (50/135), lifestyle activities (40-50%), Insulin logger (20%), integrated to health record (40%), medication log (40%) and link to glucometer (35%). |
| [32]  Canada | Evaluate a web-based coaching app | Qualitative | Patients with T2DM | 16 patients divided in 4 groups  Male: 10 | G1: 57  G2:59  G3: 42  Dropouts: 45 | Web-based coach app | Self-efficacy, competing priorities, previous behaviour change and beliefs about web-based solutions interact to determine engagement and impact on the clinical outcomes. | Demographics: newly diagnosed patients were keen to engage with mobile technology to help support self-management and have proactive information-seeking behaviour; pre-existing accountability to self; self-management support by family and nurses.  Apps features and functionalities: ability to visualise their entered data; data tracking e.g. diet, stress and blood glucose levels; feedback system; trend visualisation; real-time, nuanced performance feedback; feedback displaying desired outcomes “drew attention to positive behaviours”.  Barriers to using apps: uncertainty about the benefits of using apps; perceived data entry as burden; duplication of current logbook methods; did not perceived the technological advancement as a relative advantage; feeling overwhelmed with the idea of change; want to give-up and struggle to cope. |
| [33]  Canada | Examine patients’ attitudes and intentions to adopt assistive technologies in T2DM self-management regimes | Cross-sectional | Patients with T2DM | 44  Male: 25 | 58.7 ± 11.02 | Assistive technologies including mobile apps for diabetes self-management | Positive attitudes about using Internet and smartphone apps for self-management. Younger age was associated with more intention to use this technology. | Demographics: younger patients had more favourable attitudes toward using mobile apps; Patients who were recently diagnosed with diabetes had more favourable attitudes toward using smartphones in management.  Apps features and functionalities: glucose tracking options (90%); dietary planning options (87.5%); communicate with HCPs (85.7%). |
| [42]  Germany | Identify factors associated with app use | Cross-sectional | Patients with CVD and DM | 1500  Male: 848  DM: 681  DM and CVD: 542 | Patients with DM: 54.91 ± 8.67  Patients with DM+ CVD: 55.68± 8.25 | mHealth apps | Health app users were younger, more likely female, better educated, higher e-Health literacy and reported more physical activity; physical activity and weight loss being the most prominent target behaviours. | Demographics: health apps users were younger, more likely female, higher level of education and high level of e-health literacy.  Apps features and functionalities: behaviours targeted by apps: physical activity (289/402); Nutrition (146/402); weight loss (150/402); measuring BP, BG and step counter (184/402); sleep control (123/402); see patient’s chart or lab (21/402); relaxation (30/402); records on disease (61/402); contact doctor (23/402); medication adherence (34/402); health information (28/402); promoting goal setting (224/402); providing feedback on performance (199/402); providing opportunities for social comparison (54/402); planning social support (32/402); relapse prevention (23/402); training emotional control (37/402) |
| [22]  USA | Assess use and interest in mHealth technology for diabetes self-care | Cross-sectional | Patients with T1DM and T2DM | 60  Male: 21 | G1: 63.1±2.4 G2: 61.9±2.2* | mHealth in general including mobile apps | Patients interested in health-related text massages, mHealth apps for diabetes self-care; Younger patients were more likely to be interested in using applications to manage their diabetes. | Demographics: interesting in health-related text messages and apps was higher in smartphone users;  Younger patients were more likely to use internet and use mobile apps to help them manage their diabetes and receiving text messages.  Apps features and functionalities: Diet, meal ideas and calorie counter (20/60), blood glucose tracking (23/60), blood pressure tracking (26/60), exercise tracking and reminders (21/60), weight tracking (20/60), pedometer (21/60), medication alarm (16/60), and doctor’s visit notes (18/60).  Barriers to using mHealth: Prefer in-person diabetes management services. |
| [38]  Australia | Acquire a greater understanding of the perceived useful features, facilitators and barriers to app usage for DSM in a rural population | Qualitative | Patients with T2DM | 30  Male: 16 | 30-39yr: 1  40-49yr: 7  50-59yr: 5  60-69yr: 12  70-79yr: 5 | DSM mobile apps | Features and functions perceived as useful included visual presentation of trends, encouragement of self-motivation, convenience and user-friendly designs, however, barriers included a lack of awareness and inadequate internet access in rural areas, technology and health literacy. The findings may guide app developers in improving app design and usability. | Demographics: personal and social factors: health literacy and technical literacy.  Facilitators to using apps: App perceived to be useful; app user-friendly; convenience; apps connected to glucometer; calculating content of food; recommended by HCPs.  Barriers to using apps: technological issues e.g. app failing to work as intended; not being user friendly; difficult to navigate.  feeling they did not need an app; Not knowing about available apps; not having thought of using apps for DSM before; diabetes condition not bad; current care being sufficient; require internet connectivity. |
| [46]  Rwanda | Assess the needs and expectations of diabetic patients for mHealth supported DSM in order to develop patient-centred smartphone apps | Qualitative | Patients with T1DM and T2DM | 21  Male: 10  T2DM: 10 | 35.29 | mHealth app supported DSM | The expectations and needs of patients with diabetes is the strong focus on functions and design of future app. | Apps features and functionalities: App provided information about diabetes generally, different types of diabetes, medications; side effects of medications; medical parameters e.g. Bp, BG; medical devices; updates in diabetes; foot problems; nutrition; physical activity; alcohol consumptions; emotional and social support; motivational content; visuals; reminders; data security.  Barriers to using apps: lack of digital knowledge could be barrier to use apps. |
| [23]  USA | Determine what factors may be assocciated with apps use by clinicians working in diabetes | Cross-sectional | Clinicians working with patients with DM and obesity | 583 clinicians  Male: 9 | 22-29yr: 50  30-39yr: 75  40-49yr: 87  50-59yr: 155  60-69yr: 111  70-73yr: 4 | Mobile apps | Positive attitudes toward using apps; most agreed that apps were superior to traditional methods for patients to track dietary intake and physical activity. | Demographics: clinicians with master’s degree and registered dietitian nutritionists were most likely to be app enthusiasts.  Reasons for recommending apps to patients: tracking diet better (447/583), apps were portable (436/583), apps provided immediate feedback (425/583), tracking physical activity better (418/583), helped in making better food choices (367/583).  Clinicians believes that apps were effective for: assessing dietary intake (425/583), physical activity (408/583), blood glucose (335/583), health goals (322/583).  Reasons to recommend apps instead of other methods for dietary intake/physical activity: accountability (7 times), helpful for low-literacy patients (3 times), easier for carbohydrate counting (4 times), and low cost (2 times).  Barriers to using or recommending apps: apps not being user-friendly (16 times), lacking accuracy (14 times), patient’s access to apps (13 times), not being universal to all client (9 times), being time-consuming to use (8 times), and not containing desired features (6 times).  Decision to recommend apps based on: patients’ access to smartphone (413/583), patient was already using apps successfully (361/583), Patient’s overall level of literacy (274/583), patient’s health literacy level (202/583).  Experiences in using apps personally and professionally: clinicians need more training because they were not technologically savvy (10 times). |
| [36]  UK | Determine the use and recommendation of mHealth apps by CP and diabetic patients | Cross- sectional and qualitative | CP  Patients with T2DM | CP  95  Male: NP  Patients  9  Male: NP | CP: NP  Patients: NP | Mobile health app | More than half of the pharmacists were aware of health apps and recommended them to patients; Patients were interested in using diabetes apps and reported the facilitators and barriers toward using apps. | CP:  Awareness of the existence of mobile health apps (53/95), CP recommended apps for patients (32/53).  Patients:  Facilitators to using apps: very beneficial helpful to manage their condition (3/9).  Apps features and functionalities: reminders to manage their diabetes (6/9), use visual aids for education and monitoring (3/9), identifying effect of food on their blood glucose (2/9), having forum for social interaction (8/9), sharing data with HCPs (6/9).  Barriers to using apps (non-users’ opinion): lack of awareness of the existence of apps, time constraints, finding such apps pointless or an inconvenience, limited available functions, not frequently used due to plain forgetfulness.  Barriers to using apps (users’ opinion): time constraints (5/7), privacy and security (1/7). |
| [37]  UK | Understand the impact of using Web based and mobile technologies to support the management of T2DM | Qualitative | Patients with T2DM | 15  Male  5 | 55.4 ± 10.68 | Web-based and mobile technologies including mobile apps | Web-based and mobile technologies could offer one solution to support patients with T2DM. The findings demonstrate how patients engaged in technology use multiple apps to optimise DSM. | Apps features and functionalities: recording and monitoring BG; peer communication and support; sourcing or logging nutritional or exercise information; sourcing general information about diabetes. Stress management; diet; weight loss; managing medications, managing depression; linking to glucometer; alarm, reminder, notification system. Use of graphs to present data; goals setting; real-time feedback; gamification techniques; user-friendly app.  Barriers to using apps: complex, inconveniences including difficulties in entering data; cost of buying new equipment that link wirelessly to an app. |
| [45]  Denmark | Explore the perspectives and needs of people with T2D for a tailored digital intervention for improving diabetes self-managment | Qualitative | Patients with T2DM | 12  Male: 7 | 58.75 | Digital intervention including mobile apps | People with T2DM are unprepared for digital intervention for disease management; the main barriers to adherence with digital solutions were: experiencing diabetes distress and the need for human interaction. | Barriers to using digital intervention: older patients lacked technical literacy and rarely used the internet; technology represented ways of restricting freedom; the fear of control (big brother is watching, feeling frustration (my smartphone doesn’t understand me); want human interaction and personal help to solve their health problem. |
| [24]  USA | Identify patients who have and are willing to connect to the app, and examine barriers to the use of the technology | Retrospective cohort | Patients with chronic conditions including DM | 503  Male: 255 | 18-34yr: 169  35-49yr: 200  50-64yr: 100  65-79yr: 32  80 and over: 2 | Apple’s HealthKit that is linked with electronic medical records | App users had a younger age distribution than non-users; app users rarely have extremely high HbA1C; and level of education was reported as a barrier to using the app. | Demographics: The registration in the app is higher among young patients.  Facilitators to using apps: “tech-savvy”; iPhone savvy patients who understand their iPhone well enough to load app/ register in apps. |
| [25]  USA | Identify the barriers to adoption and sustained use of H360x technology in care settings | Qualitative | Patients with DM | 287  Male: 86 | 62.1 ± 13.18 | Health360x (H360x) is a mobile app and social platform that integrates DSM and decision support | Barriers to adoption were inability to use the internet and concerns about privacy and security of health data. | Barriers to using the app: Patients’ anxiety about use of the internet and discomfort with putting health information on the internet. |
| [26]  USA | Understand the perceived barriers, benefits and facilitators among adults with T2DM regarding using mobile apps | Qualitative | Patients with T2DM | 18  Male: 5 | 54 ±12.7 | Free mobile app available ion the App Store or Google Play for diabetes management and behaviour change | Main barriers reported were information gap, technical illiteracy and satisfaction with traditional tools; patients will use the app if it is easy to use, has specific features e.g. tracking, educational and provides customised information. | App features and functionalities: intuitive and easy to use; tracking features; diabetes information e.g. glycaemic index information, diet, tips, diabetes recipes and recent research findings; personalised or customised information including customised feedback and recommendation based on tacked data; reminders; goal setting, visual presentation of data to show history, trends, and progress on long-term basis; sharing data online with HCPs.  Barriers to using apps: not aware that these tools were available; technology illiteracy; satisfied with traditional tools; not recommended by doctors; not want to be accountable for their behaviours; require time and effort to use apps; complexity of entering detailed information. |
| [34]  Canada | Explore the use of a mobile app and its effects on motivation for health behaviour change after RCT | Qualitative | Patients with T2DM | 11  Male: 2 | Male: 63.5 ± 4.9  Female:  55.8 ± 8.8 | Mobile app for health coaching | Patients reported some features that emphasize the benefits of using mobile apps e.g. blood glucose monitoring, diet and exercise; user-friendly apps with reminders and remote feedback were also reported. | App features and functionalities: user-friendly; exercise tracking; food tracking (via photo-journaling), health coach communication and self-generating/coach generated reminders; dietary feedback; reminder messages; immediate feedback.  Barriers to using apps: learning to use the app is a challenge. |
| [41]  KSA | Investigate patterns of usage pretaining to health apps among diabetic patients in KSA as well as patients preference and challenges of using health apps | Cross-sectional | Patients with DM | 355  Male: 253 | <18yr: 2  18-24yr: 47  25-44yr:  110  45-64yr: 160  >65yr: 33 | Mobile health app | Around one third of diabetic patients reported using health apps. Patients preferred to follow up BG levels, body weight, exercise and calorie intake. The main barriers reported are patients don’t want to know about their health or don’t know how to start using apps. | Demographics: Health apps usage was significantly associated with female gender, younger participants (<44 years), unmarried, and higher educational level.  Facilitator to use: Training is needed to use apps;  Apps features and functionalities: ease to use; BG measurement; monitoring body weight; total calories intake; medication reminders, tracking exercise accomplishments; pedometer; BP measurement; foot pressure measurement; communication with HCPs .  Barriers to using apps: not recommended by HCPs; health apps were difficult to understand; patients don’t want to know about health issues; patients unsure how to start to use apps; it takes too much time to enter data; app would gather too much personal data, hidden or high cost; lack of interest; privacy concerns. |
| [27]  USA | Explore behaviours and perceptions about mobile phone-based apps for health among individuals with chronic conditions | Cross-sectional | Patients with chronic conditions including DM | 1604  Male: 809  Diabetes: 163  Male: 84 | 41.4 ±16.5 | Mobile health app | Individuals with poor self-reported health and low rates of physical activity, arguably those who stand to benefit most from health apps, were least likely to download and use these health tools. | Facilitators to using apps: improves health  Apps features and functionalities: tracking activity or exercise, helps track/improve diet, aids weight loss, tracks a health measure/ helps relaxation (6/163).  Barriers to using apps: does not help improve health (lack of perceived benefit). |
| [43]  Germany | Explore the factors influencing the acceptence of diabetes apps among patients aged 50 or older | Qualitative | Patients with T1DM and T2DM | 32  Male: 16  T2DM: 21 | 68.8 ± 8.2 | Mobile app | Lack of additional benefits and ease of use were the key factors for the acceptance of diabetes apps; diabetes app should be individually adapted to overcome the variety of patients’ needs. | App features and functionalities: feedback on measured values e.g. blood glucose, definition of thresholds/highlighting deviating values, reminder feature for measurement/medication, up-to-date information on disease/therapy/ medication, recording and management of values (HbA1c, weight), overview of all values on a single page or table, overview of measured values in general, automatic data transmission from measurement device/insulin pump to app, data transmission to attending physician, reference book of nutritional values on dishes in restaurants, grocery stores, nursing homes, preparation of the contents of the training received immediately after diabetes diagnosis.  Barriers to using apps: apps does not fit personal needs in diabetes management, relevant functions/contents not available such as management of polypharmacy or comorbidity, functions/contents not reasonable/relevant, apps do not provide additional benefits, handling is too complicated/ time consuming, storage process/single steps not intuitively understandable. |
| [28]  USA | Study if and how mobile app functions to improve T2DM management would be desired in low income African American community | Qualitative | Pre-diabetes,  T2DM patients, family member, friends, HCPs | 78  HCPs: 20  Pre-diabetic or T2DM patients: 31 |  | Social support mobile apps for T2DM | Participants perceived apps as a tool aiding DSM. | Apps features and functionalities: social support through the app could motivate exercising, nutrition e.g. food choices; use of reminders for medications and medical visits ; find companionship to exercise or visit the doctor; monitor each other through the app e.g. check BG, medications; sharing successful strategies and personal stories e.g. diabetes foods, recipes, dealing with difficult situation; developing a system of automatic alerts to be sent to emergency contacts; information about diabetes management. |
| [29]  USA | Survey adults with T2DM regarding self-monitoring behaviours, including mobile application  use | Cross-sectional | Patients with T2DM | 96  Male: 45 | 60.74 ± 11.03 | Mobile app for T2DM | The most common app tracked behaviour was physical activity, then weight and diet; despite numerous available mobile health-tracking applications, only a few T2D adult patients from the study sample used them. | Apps features and functionalities preferred by patients who already used apps: tracking activity, diet, weight, medications, blood glucose, sleep and blood pressure. |
| [30]  USA | Evaluate the usability of mobile apps for self-management of diabetes and hypertension | Qualitative | Patients with HTN and/or diabetes  HCPs  Research assistants | 8 patients  3 HCPs  2 research assistants  Male: NP | NP | Mobile app for DSM and HTN | Effective use of mHealth tools requires a good fit between the app, the users’ eHealth literacy, the treatment approach, staff time and reimbursement for services. | Patients:  Apps features and functionalities: easy/complex to use, quicker/slower to get responses; app can be used to request medication refill; app should be simple and easy; should connect to electronic health records; ease of contact with providers; should include notifications if there is a message from healthcare providers; the app has a social media component; and includes medication reminders.  Barriers to using apps: patients rely on their healthcare providers as they “tell me what to do”; don’t know how to download a free app; concerns about the privacy of health information on the app.  Staff:  Barriers to using apps: patients did not have access to technology, were not comfortable with technology; do not use phone other than for calls; forget app’s password; lack of time; downloading the app takes a long time; loss of interest if patient couldn’t download the app right away; not enough time for the staff to explain the app during patient’s visit; patients did not want to wait after their visit to show them how to use the app; the app did not fit the nurses’ workflow; nurses reported a lot of uneasiness about messaging with patients; immediate response of nurses could not be provided; patients did not use the app as directed; health is not the patients’ first priority, app was asking them to do too much. |
| [48]  Norway | Obtain in-depth understanding of user’s acceptability of mobile apps for DSM | Qualitative | Patients with T2DM | 24  Male: 11 | 61 | DSM mobile apps | Users’ acceptability of a mobile app for DSM differed and practical and social acceptance are important. | Facilitators to using apps: perceived usefulness of apps.  Apps features and functionalities: Ease of use; accessibility of the app; automatic transfer of BG levels to the app; including graphs and charts of data; reminders; automatic tracking; sending data to HCPs; BG management; diet and exercising plans; tailored feedback; educational information  Barriers to using apps: cost of time and effort to use apps; manually entering of data; stressful constant reminders; poor health that hinders physical activity; lack of usability of smartphone and apps; unable to interpret data patterns in the app; satisfaction with the current management tools. |
| [39]  Australia | Explore the use of mobile apps among adults with T2DM | Cross-sectional | T2DM patients | 796  Male: 475 | 60 ± 9 | Mobile app | A minority of adults with T2M use mobile apps; exercise-tracking app was the most frequently used app. | Demographics: App users were younger; were diagnosed more recently; and exhibit a higher frequency of blood glucose self- monitoring.  Apps features and functionalities: carbs counting, recording blood glucose levels, tracking physical activity, tracking weight, Insulin dose calculation, monitoring mood/well-being, social support, hypos/hypo symptoms.  Barriers to using apps: apps could not help in diabetes management, no good app was found yet, do not know how to use apps, lack of awareness of app existence. |
| [49]  China | Provide information regarding the optimal design of diabetes apps | Cross-sectional | DM patients and diabetologists | Patients  1276  Male: 642  T2DM: 733  Diabetologists  608  Male: 197 | Patients  Overall: 41.3± 18.5  T2DM: 52.2±12  HCPs  Diabetologists:  <30yr: 99  30-39yr: 274  40-49yr: 162  50-59yr: 67  ≥60: 6 | Apps for DSM | The awareness and use of DSM apps among patients and diabetologists are low; User engagement in designing apps is needed to ensure that apps are suitable for targeted groups; public awareness of diabetes apps should increase and relevant policies and regulations are needed to support doctors’ use of apps to manage patients. | Patients  Demographics: app users tend to be younger patients, with high income and a high education level.  Apps features and functionalities: Diabetes diaries; Doctor-patient communication; diabetes education knowledge; peer support; insulin dose calculator; abnormal blood sugar reminder; blood sugar test reminder;  Barriers to using apps: limited time; complicated operations; ineffectiveness for glycaemic control and cost.  Recommendations  38/197 app users used apps based on recommendation by HCPs; 55/197 used apps based on recommendation by other patients; 30/197 used apps recommended by media; and 54/197 used apps randomly.  Diabetologists  Recommendations: 246/608 of diabetologists recommended apps to patients; and 153/608 used apps to manage their patients.  Apps awareness increased with diabetologists’ age.  The highest recommendation rate and usage were found in the 40 to 49 age group and then, recommendation likelihood decreased with age.  Recommendation and usage rate among doctors in tertiary hospitals were higher than those in secondary care or lower.  Factors influencing diabetologists’ recommendations of apps: Not knowning of suitable apps (296/608); not knowing of the exsitence of DSM apps (212/608); no time to recommend apps (182/608); no evidence demonstrating their effectiveness (90/608); and no effect on blood sugar (47/608).  Barriers to using apps to manage patients: Limited time (280/608); legal issues (129/608); patients’ distrust (108/608) of apps; uncertainty in how to bill patients (66/608).  Preferred apps features and functionalities: Diabetes diaries; and doctor-patient communication. |
| [31]  USA | Identify opportunities to support patients with multiple chronic conditions through new and enhanced eHealth technology | Qualitative | Patients with MCCs including DM | 53  Male: 39 | 59 ± 11 | e-Health including health-related mobile applications. | The most frequent practice was to search for health information, communicate with HCPs, track medical information and track medications. | Apps features and functionalities organising medication regimens, facilitating communication with providers, providing information about their multiple conditions, task management e.g. printing monthly activities, detecting potential drug interactions, accessing new research findings, providing a forum to connect with other patients, enabling health records storage, tracking, sharing and offering opportunities to consult remote specialists and coordinate care with local providers, and integrating information from different healthcare systems (e.g. appointment reminders). |

CP: community pharmacists; NP: not provided; T1DM: type 1 diabetes mellitus; T2DM: type 2 diabetes mellitus; DM: diabetes mellitus; G: group; HCW: Healthcare workers; HCPs: Healthcare professionals; HTN: hypertension; DSM: diabetes self-management;

*Patients characteristics were stratified by practice site into two groups.
